# Regulation of Constitutive Interferon-Stimulated Genes (Isgs) in Tumor Cells Contributes to Enhanced Antitumor Response of Newcastle Disease Virus-Infected Tumor Vaccines

**DOI:** 10.3390/cancers10060186

**Published:** 2018-06-06

**Authors:** Mai Takamura-Ishii, Takaaki Nakaya, Katsuro Hagiwara

**Affiliations:** 1 School of Veterinary Medicine, Rakuno Gakuen University, 582 Bunkyodai, Ebetsu, Hokkaido 069-8501, Japan; s21441002@g.rakuno.ac.jp; 2 Department of Infectious Disease, Kyoto Prefectural University of Medicine, Kajii-cho, Kawaramachi-Hirokoji, Kamigyo-ku, Kyoto 602-8566, Japan; tnakaya@koto.kpu-m.ac.jp

**Keywords:** Newcastle disease virus, tumor, vaccines, ISG, ruxolitinib

## Abstract

Newcastle disease virus (NDV) is an oncolytic virus. As immunogenicity of tumor cells is enhanced by NDV infection, recombinant NDV-infected tumor vaccines (rNDV-TV) are effective methods for inducing specific immunity. However, several tumor cells resist NDV infection, and tumor specific immunity is not sufficiently induced by rNDV-TV. Therefore, we clarified the factor contributing to the suppression of NDV infection and attempted to improve rNDV-TV. Initially we investigated the correlation between the NDV infection rate and interferon-related gene expression in six *murine* tumor cell lines. A significant negative correlation was observed between the constitutive gene expression of Interferon-stimulated genes (ISGs) and NDV infectivity. The NDV infection rate was examined in each tumor cell treated with the Janus kinase (JAK) inhibitor ruxolitinib (Rux). Furthermore, we evaluated the Th1 response induction by Rux-treated rNDV-TV (rNDV-TV-Rux). In Rux-treated tumor cells, *Oasl2* gene expression was significantly decreased and viral infectivity was increased. In immunized mice, the number of CD8^+^ cells, and those expressing the IFN-γ gene, were significantly increased as compared with Rux-untreated rNDV-TV. The infectivity of the virus was dependent on the degree of ISGs expression in tumor cells. To remedy for this problem, rNDV-TV-Rux was expected to have a Th1 immune response.

## 1. Introduction

Newcastle disease virus (NDV) is characterized by selective viral replication of tumor cells [1]; this virus is known to be oncolytic i.e., it kills infected tumor cells and oncolytic therapy with NDV has been studied [2]. Moreover, in addition to direct tumor cytotoxicity due to its oncolytic properties, NDV-infected tumor cells were reported to induce the maturation of dendritic cell by type I interferon (IFN) [3,4], activation of antigen specific T cells [5], immunostimulatory effects such as the activation of NK cells by hemagglutinin-neuraminidase (HN) protein expressed on an infected cell surface [6], and increase in MHC class I expression [7]. Currently, an NDV-infected tumor vaccine (NDV-TV), using NDV as an adjuvant of tumor cell vaccine, has been studied and clinically tested in humans with mammary adenocarcinoma, ovarian cancer [8], colon cancer [9], squamous cell carcinoma of the head and neck [9,10], and glioblastoma multiforme [11]. These reports indicated an effective anti-tumor response induced by NDV-TV. 

**Figure 1:**
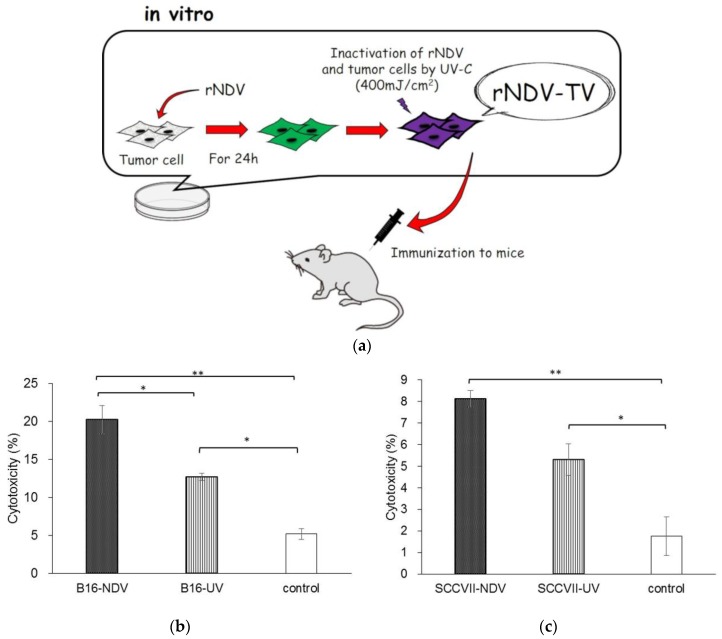
Newcastle disease virus (NDV)-infected tumor vaccines (NDV-TV) model and induction of antitumor response by rNDV-TV. (**a**) The schematic of NDV-TV model was shown. The tumor cells were infected with rNDV in vitro, irradiated by ultraviolet for inactivation of rNDV and tumor cells. Then rNDV-TV was administered to mice as immunogen. rNDV-TV (*n* = 3), UV-TV (ultraviolet irradiated tumor vaccine) (*n* = 3), or each medium (*n* = 2) were administered to mice and splenic mononuclear cells (SMCs) were co-cultured with UV-irradiated tumor cells for 5 days. After cytotoxic T cell (CTL) induction, SMCs were harvested and co-cultured with target tumor cells for 24 h. The cytotoxicity was measured using quantifying lactate dehydrogenase (LDH) in the supernatant (* *p* < 0.05, ** *p* < 0.01). rNDV-TV and UV-TV were prepared using (**b**) B16 and (**c**) SCC VII. Medium was administered to the control mice.

Previously, we generated a green fluorescent protein (GFP)-expressing recombinant NDV (rNDV). The virus induced cell death only in infected cells without producing any infectious virus particles, because the viral F protein cleavage site amino acid sequence was transformed (G-R-Q-G/S-R; L) [12]. Furthermore, we examined the induction of anti-tumor response induced by the rNDV infected tumor vaccine (rNDV-TV). rNDV-TV was prepared as tumor cells were infected with rNDV in vitro, then irradiated by UV for inactivation of both rNDV and tumor cells. The rNDV-TV was administered to mice as an immunogen (Figure 1a). In murine melanoma, the mice inoculated with rNDV-TV had significantly suppression of tumor metastasis compared to the control. And, the rNDV-TV vaccinated group survived longer than the control group. [13]. Also in murine fibrosarcoma, the mice inoculated with rNDV-TV induced an effective anti-tumor response and complete tumor exclusion [14]. These experiments revealed the induction of an anti-tumor response by rNDV-TV. However, the results indicated that there were differences in the anti-tumor effect of rNDV-TV between used tumor cell lines.

Therefore, in this study we focused on the difference in the anti-tumor response of rNDV-TV and the rNDV infectivity between several murine tumor cell lines. Moreover, to improve the anti-tumor response of rNDV-TV, the possibility of a new therapeutic strategy was investigated.

## 2. Materials and Methods

### 2.1. Animals

Specific pathogen-free C57BL/6 and C3H/HeN *murine* strains were obtained from CLEA Japan, Inc. (Tokyo, Japan). Mice were maintained in a sterile isolator and treated according to the Laboratory Animal Control Guidelines of the National Guidelines for the Care and Use of Laboratory Animals (Rakuno Gakuen University approval number: VH14A4).

### 2.2. Cell Culture and Virus Production

The *murine* fibrosarcoma cell line WEHI164 (laboratory stock cell line), mammary tumor cell line BALB-MC (derived from BALB/c mice, #JCRB0233.0, JCRB cell bank, Ibaraki, Japan), melanoma cell line B16 and Lewis lung carcinoma cell line 3LL (derived from C57BL/6, #JCRB0202 and # JCRB1348, JCRB cell bank, Ibaraki, Japan), squamous cell carcinoma SCC VII (provided from Dr. Inanami, Hokkaido University, Sapporo, Japan), and bladder carcinoma MBT-2 (derived from C3H/HeN, # IFO50041, JCRB cell bank, Ibaraki, Japan) were used. WEHI164 and 3LL were cultured in RPMI-1640 medium (Sigma-Aldrich, St. Louis, MO, USA), B16 was cultured in Dulbecco’s modified Eagle’s medium (DMEM; Sigma-Aldrich, St. Louis, MO, USA), and the others were cultured in Minimum Essential Medium (MEM; Gibco, Waltham, MA, USA). The cells were cultured in each medium supplemented with 5% fetal bovine serum (FBS; Gibco, Waltham, MA, USA), 200 U/mL penicillin (Meiji, Tokyo, Japan), and 200 μg/mL streptomycin (Meiji, Tokyo, Japan) at 37 °C in a humidified atmosphere with 5% CO_2_. GFP-expressing rNDV was generated as previously described [12] and proliferated in embryonated chicken eggs. rNDV was collected from the allantoic fluid, and the number of focus-forming units (FFUs) was determined.

### 2.3. Preparation of rNDV-TV, Ultraviolet Irradiated Tumor Vaccine (UV-TV) and Ruxolitinib-Treated rNDV-TV (rNDV-TV-Rux)

The tumor cells (10^5^ cells/well) were cultured with each medium supplemented with 5% FBS in 6-well plates. For rNDV-TV-Rux preparation, the cells were cultured in the appropriate medium, containing 1 μg/mL of janus kinase (JAK) inhibitor, ruxolitinib (Rux; Cayman Chemical, Ann Arbor, MI, USA), for 20 h before rNDV infection. Then, the cells were infected with rNDV using a multiplicity of infection (MOI) of 2 and cultured at 37 °C for 24 h in a CO_2_ incubator. rNDV-infected tumor cells were harvested with ethylenediaminetetraacetic acid and phosphate-buffered saline (EDTA-PBS), then inactivated with UV irradiation (400 mJ/cm^2^). After washing with PBS, the cells were resuspended at 5 × 10^5^ cells/mL in the correct medium. UV-TV was prepared by UV irradiating (400 mJ/cm^2^) each tumor cell, those without rNDV infection were also prepared.

### 2.4. Immunization Protocol

Each vaccine was administered intraperitoneally to mice at 10^5^ cells/mouse four times at weekly intervals. For control, 0.2 mL of each cell culture medium was administered intraperitoneally to mice. 

### 2.5. Cytotoxicity Assay

Spleen cells were collected from immunized mice, and splenic mononuclear cells (SMCs) were separated by density gradient centrifugation using Ficoll–Conray solution (d = 1.088). To induce the cytotoxic T lymphocytes (CTL), the SMCs were cultured with inactivated tumor cells for 5 days at an effector–target ratio (E:T) of 4:1. After a 5-day pre-stimulation with the tumor cells, effector SMCs were separated by density gradient centrifugation with Ficoll–Conray solution. SMCs were washed with PBS and co-cultured with each tumor cell at an E:T of 20:1 for 24 h. Cytotoxicity was determined by lactate dehydrogenase (LDH) release using a Cytotoxicity Detection KitPLUS (LDH) (Roche Diagnostics, Basel, Switzerland). The cytotoxicity rate was calculated as follows: Cytotoxicity (%) = (Experimental LDH − Effector spontaneous LDH − Target spontaneous LDH)/(Target maximum LDH − Target spontaneous LDH) × 100 (rNDV-TV and UV-TV group, each *n* = 3; control group, *n* = 2).

### 2.6. Viral Infection

The tumor cells (5 × 10^5^ cells/well) were cultured with each cell adopted medium containing 5% FBS in 6-well plates. After washing with FBS-free medium, the cells were adsorbed with rNDV using a multiplicity of infection (MOI) of 2 for 1 h in a CO_2_ incubator. Then, the cells were cultured in medium with 5% FBS at 37 °C for 24 h in a CO_2_ incubator, and observed with a fluorescence microscope (Zeiss, Oberkochen, Germany). The infection rate was calculated as follow: Infection rate (%) = Tumor cells expressing GFP/Total tumor cells × 100.

### 2.7. RNA Extraction and cDNA Synthesis

Total RNA was extracted with the RNeasy Mini Kit (Qiagen, Hilden, Germany) according to the manufacturer’s instructions. Sample were lysed with 350 μL of RLT buffer, and total RNA was eluted with 50 μL of RNase-free water through the RNeasy Mini spin column. 

For cDNA synthesis, Transcriptor First Strand cDNA Synthesis Kit (Roche Diagnostics, Switzerland) was used. Briefly, 1 μg of total RNA was reverse-transcribed using oligo (dT) primers and incubated for 60 min at 50 °C after denaturation. The reverse transcriptase was then inactivated by 5 min heating at 85 °C.

### 2.8. Quantitative Polymerase Chain Reaction (qPCR)

qPCR was carried out with a Light Cycler 2.0 (Roche Diagnostics, Basel, Switzerland), QuantiTect SYBR Green Kit (Qiagen, Hilden, Germany) for the detection of genes. The target genes were normalized with glyceraldehyde-3-phosphate dehydrogenase (GAPDH). The amplification conditions consisted of 45 cycles of 94 °C for 15 s, 60 °C for 30 s, and 72 °C for 15 s. The sequences of primers used in qPCR were shown in supplementary Table S1.

### 2.9. JAK Inhibition

To confirm adequate quantity of Rux for suppression of JAK in tumor cells, tumor cells were incubated with various dose of Rux. Tumor cells (10^5^ cells/well) were cultured with each cell adopted medium containing 5% FBS in 24-well plates for overnight. Then, the cells were washed with FBS-free medium once, and exchanged the medium containing from 0 to 1.0 μg/mL of ruxolitinib, and incubated for 20 h. 

### 2.10. Cytokine Expression

To monitor cytokine gene expression after tumor stimulation, the separated SMCs from immunized and non-immunized mice were used as effector cells and the tumor cells were used as target cells. Effector cells and target cells were co-cultured at an E:T of 20:1 for 6 h at 37° C. Then, SMCs were lysed with 350 μL of RLT buffer (Each group, *n* = 5). The samples were extract RNA for determine the gene expression.

### 2.11. Flow Cytometry

To monitor lymphocyte subsets in SMCs from each groups of mice, the cells were incubated with CD4-FITC mouse (Milteny Biotec, Bergisch Gladbach, Germany) and rat anti-mouse CD8a/Lyt-2-PE (Beckman Coulter, Brea, CA, USA), or FITC anti-mouse CD3 and PE anti-mouse CD19 (Bio Legend, Cromwell, CT, USA) for 30 min at room temperature. The cells were then washed with PBS twice, treated with 0.5% formalin-PBS, and used for flow cytometry analysis with a Coulter Epics XL (Beckman Coulter, Brea, CA, USA).

### 2.12. Statistical Analyses

Differences between two groups of data were calculated with the Student’s *t* test. Correlation analyses were performed using the Spearman’s correlation coefficient. Multiple comparison analyses were calculated with the Tukey–Kramer multiple comparison method. Values were regarded as significant at *p* < 0.05. The analyses were calculated with R version 1.0.143.

## 3. Result

### 3.1. Induction of Anti-Tumor Response with rNDV-TV

To confirm the anti-tumor response with rNDV-TV, UV irradiated tumor vaccine (UV-TV) or the medium, the cytotoxicity to two tumor cell lines, B16 and SCC VII, was investigated. The average of cytotoxicity to B16 in B16-NDV immunized mice was 20.3% (Figure 1b). This was significantly higher than that of the B16-UV (*p* = 0.04) or control group (*p* = 0.005). The average of cytotoxicity in B16-UV was significantly higher than that of the control group (*p* = 0.04). The cytotoxicity to SCC VII in SCC VII-NDV and SCC VII-UV immunized mice was 8.1% and 5.3% respectively. These were significantly higher than that of the control group (*p* = 0.001, *p* = 0.03, respectively). However, there were no significant differences between the cytotoxicity in SCC VII-NDV and SCC VII-UV immunized mice (*p* = 0.06) (Figure 1c). The results showed the difficulty to induce the anti-tumor response by rNDV-TV in some of the examined tumor cell lines. Interestingly, the cytotoxicity induced by WEHI-NDV [14] was higher than those by B16-NDV and SCC VII-NDV. It means that there is the difference of anti-tumor response induced by rNDV-TV depends on tumor cell lines. Therefore, we hypothesized that infectivity of rNDV to each tumor cell line is one of the factor to induce the anti-tumor response by rNDV-TV among tumor cell lines.

### 3.2. Infection Rate in Murine Tumor Cell Lines

**Figure 2:**
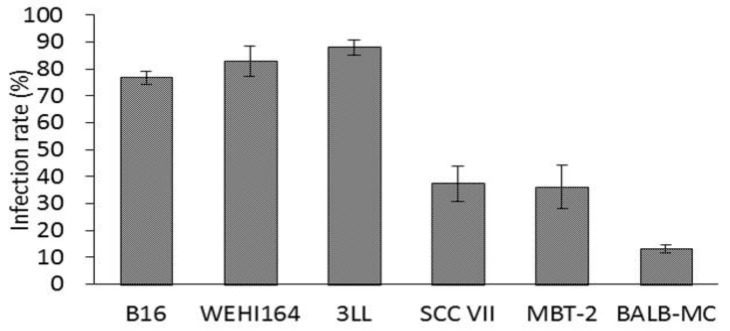
Recombinant NDV (rNDV) infection rate in murine tumor cell lines. Murine tumor cell lines were infected with rNDV (MOI of 2). The infection rate was calculated from the green fluorescent protein (GFP) expressing cell ratio in fluorescent microscopic observation.

To investigate the rNDV infectivity in tumor cell lines, the tumor cells were infected with rNDV, and the rNDV infection rate was calculated from the ratio of GFP expression cells. The rNDV infection rate in B16, WEHI164, 3LL, SCC VII, MBT-2, and BALB-MC calls were 76.8%, 83.0%, 88.1%, 37.4%, 36.2%, and 13.1% respectively (Figure 2). These showed there were difference of rNDV infectivity among tumor cell lines. Furthermore, SCCVII, which failed to sufficiently induce anti-tumor response by rNDV-TV, was rNDV low infectivity. Therefore, next, the factors of the difference in rNDV infectivity in tumor cell lines were examined.

### 3.3. The Expression of Type I IFN Related Genes in Tumor Cell Lines

To consider the difference of rNDV infectivity in tumor cell lines, the expression of type I IFN related genes induced by viral infection was evaluated. In the tumor cells before rNDV infection, the expression of retinoic acid-inducible gene-I (RIG-I), toll-like receptor 3 (TLR3), interferon regulatory factor 3 (IRF-3), myxovirus resistance (Mx) 1, Mx 2, 2′-5′-oligoadenylate synthetase (OAS) 1a, OAS1b, OAS2, OAS3, and 2′-5′ oligoadenylate synthetase-like (OASL) 2 showed a significant negative correlation with the rNDV infection rate. On the other hand, the expression of TLR3, IRF-7, OASL1, and OASL2 in tumor cells 8 h post infection showed a significantly negative correlation with the rNDV infection rate (Table 1). The detailed raw data of the type I IFN related gene expression indicated in the supplementary data (Table S2 and Figure S1). These result showed the constitutive Mx, OAS and OASL2 gene expression, one of interferon stimulated genes (ISGs), was important for rNDV infectivity of tumor cells. Then, we confirmed the improvement of rNDV infection rate by janus kinase (JAK) inhibitor which was the upper-pathway of ISGs.

### 3.4. rNDV Infection Rate in Ruxolitinib (Rux) Treated Tumor Cell Lines

**Figure 3:**
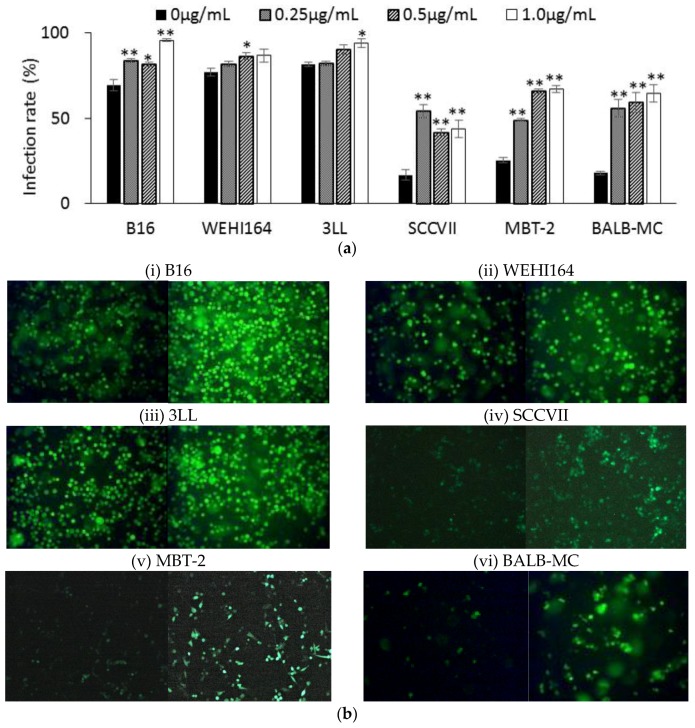
rNDV infection rate in tumor cells after ruxolitinib treatment. (**a**) Tumor cells were infected with rNDV (MOI of 2) after ruxolitinib (0~1.0 μg/mL) treatment for 20 h. The infection rate was calculated from the GFP expressing cell ratio observed via fluorescent microscopy (* *p* < 0.05, ** *p* < 0.01). (**b**) GFP expressed in (i) B16, (ii) WEHI164, (iii) 3LL, (iv) SCCVII, (v) MBT-2, and (vi) BALB-MC infected with rNDV after ruxolitinib (left: 0 μg/mL, right: 1.0 μg/mL) treatment (×100 magnification).

Since ISG expression in tumor cell lines before rNDV infection showed a significant negative correlation with the rNDV infection rate, the effect of Rux, JAK inhibitor, treatment before rNDV infection on the rNDV infectivity of tumor cell lines was examined. In the tumor cell lines with a low rNDV infectivity, SCC VII, MBT-2, and BALB-MC, rNDV infectivity was significantly increased by Rux treatment. Furthermore, in the tumor cell lines with a high rNDV infectivity the rNDV infection rate was significantly improved by Rux treatment (Figure 3a,b). These results indicated the improvement of rNDV infectivity in tumor cell lines by Rux treatment before infection. In next examination, the influence to the expression of ISGs by Rux treatment was confirmed.

### 3.5. The Expression of ISGs in Tumor Cell Lines Treated with Rux

**Figure 4:**
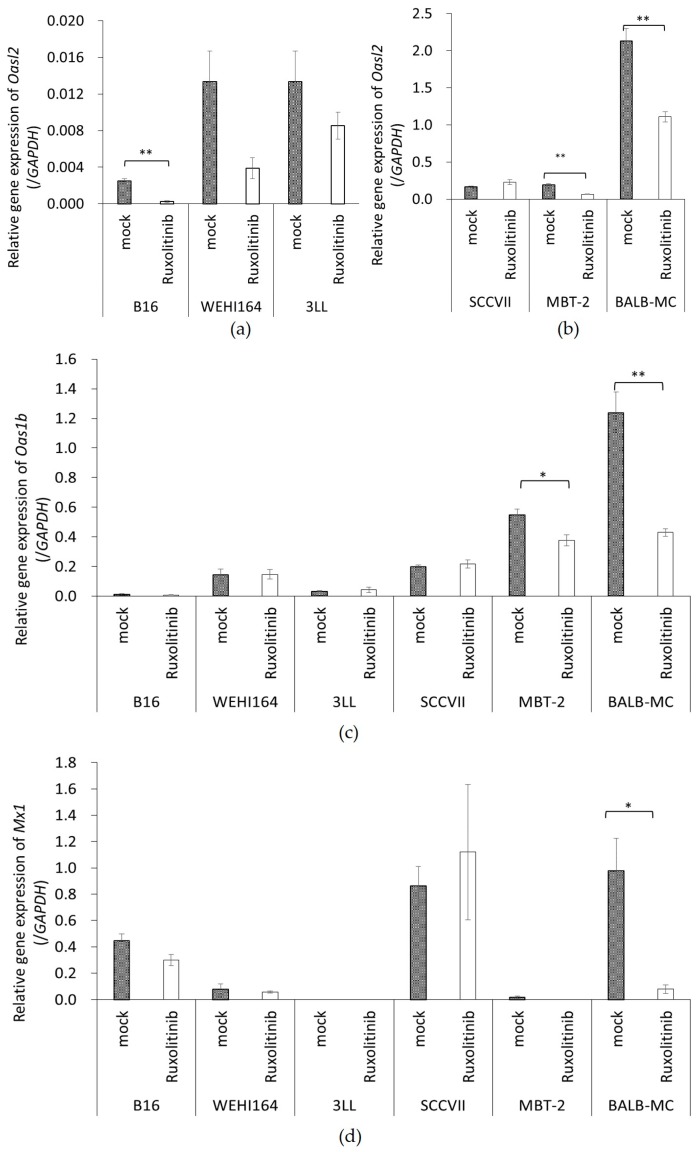
ISGs expression after ruxolitinib. Tumor cells were cultured in medium with (ruxolitinib) or without (mock) ruxolitinib for 20 h. Then, (**a**, **b**) *Oasl2*, (**c**) *Oas1b*, and (**d**) *Mx1* gene expression was quantified by qPCR. Gene expression was normalized with GAPDH (* *p* < 0.05, ** *p* < 0.01).

To confirm the effect of Rux on ISG expression, the expression of *Oas1b*, *Oasl2*, and *Mx1* was measured in Rux treated tumor cell lines. The expression of *Oasl2* was significantly decreased in B16 (*p* = 0.001), MBT-2 (*p* = 0.001), and BALB-MC (*p* = 0.005) after Rux treatment, and a decline was observed in WEHI164 (*p* = 0.001) and 3LL (*p* = 0.258) (Figure 4a). Furthermore, *Oas1b* gene expression was significantly decreased in MBT-2 (*p* = 0.04) and BALB-MC (*p* = 0.005) (Figure 4b), and *Mx1* gene expression was also significantly decreased in BALB-MC (*p* = 0.0023) (Figure 4c). Additionally, rNDV infection rate and ISG expression after Rux treatment showed a significant negative correlation (*Oasl2*: r_s_ = −0.826, *p* < 0.01, *Oas1b*: r_s_ = −0.720, *p* < 0.01, *Mx1*: r_s_ = −0.543, *p* < 0.01). These result showed ISG expression of tumor cells lines was decreased by Rux treatment and was important factor of rNDV infectivity in tumor cell lines. Then, we prepared ruxolitinib- treated rNDV-TV (rNDV-TV-Rux) which rNDV infectivity was improved by Rux treatment, and the immune response induced by rNDV-TV-Rux was confirmed.

### 3.6. Lymphocyte Subset in Murine SMC Immunized with MBT-NDV-Rux

**Figure 5:**
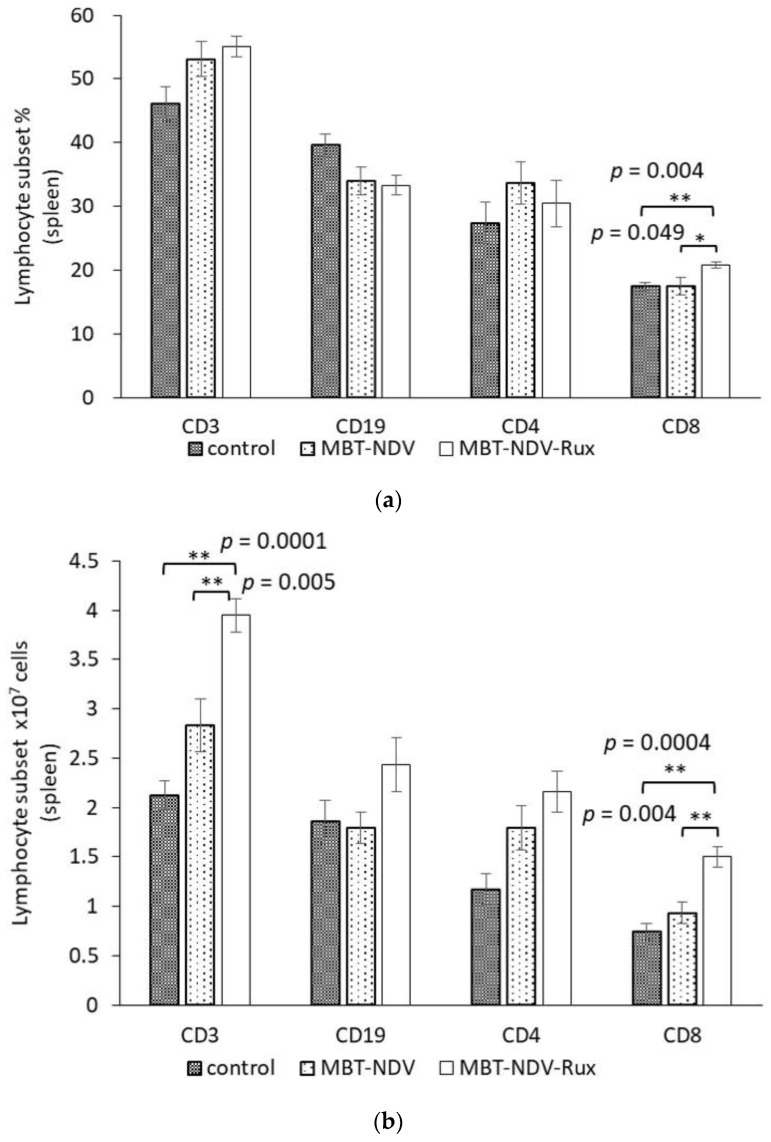
Lymphocyte subsets of SMCs after vaccination. Medium (control, *n* = 5), MBT-2 using rNDV-TV (MBT-NDV, *n* = 5), or rNDV-TV-Rux (MBT-NDV-Rux, *n* = 5) were administered to the mice. Lymphocyte subsets (**a**) ratio and (**b**) numbers of SMCs were analyzed by flow cytometry after the last vaccination (* *p* < 0.05, ** *p* < 0.01).

To confirm the immune response induced by rNDV-TV-Rux, rNDV-TV-Rux using MBT-2 (MBT-NDV-Rux) were immunized to the mice, and the lymphocyte subset in the *murine* SMC was evaluated. The ratio of CD8^+^ cells in the MBT-NDV-Rux immunized group was significantly increased compared with the control and MBT-NDV immunized groups (Figure 5a). Although there was no significant difference in *murine* bodyweight between groups, the total number of SMC in the MBT-NDV-Rux immunized group (7.22 × 10^7^ cells/head) was significantly increased compared to the control (4.65 × 10^7^ cells/head, *p* = 0.003) and MBT-NDV immunized group (5.31 × 10^7^ cells/head, *p* = 0.021). In addition, the number of CD3^+^ and CD8^+^ cells in the MBT-NDV-Rux immunized group were significantly higher than those of control and MBT-NDV immunized groups (Figure 5b).

### 3.7. IFN-γ Gene Expression in Immunized SMC with MBT-2 Stimulation

**Figure 6:**
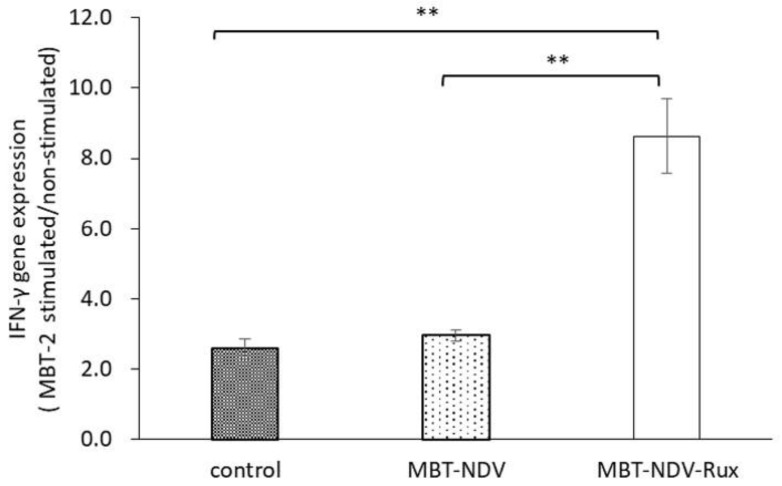
IFN-γ gene expression in MBT-2 stimulated SMCs. SMCs from immunized mice were co-cultured with MBT-2 for 4 h. Then, IFN-γ gene expression of the SMCs were analyzed by qPCR. The gene expression was normalized with glyceraldehyde-3-phosphate dehydrogenase (GAPDH), and the comparison of gene expression upon stimulation against unstimulated was calculated (** *p* < 0.01, each group *n* = 5).

To confirm Th1 immune response to MBT-2 in immunized SMC, the IFN-γ gene expression in response to MBT-2 stimulation was calculated. The ratio of IFN-γ gene expression in the MBT-NDV-Rux immunized group was significantly increased compared with the MBT-NDV and control groups (Figure 6). This result showed the induction of Th1 immune response by rNDV-TV-Rux.

## 4. Discussion

In this study, we investigated the difference of rNDV infectivity in various tumor cell lines, and its influence on rNDV-TV. Then, we examined the anti-tumor response induced by rNDV-TV-Rux as a novel treatment for rNDV infectious tumor cells.

In B16-NDV immunized mice, the cytotoxicity in tumor cells was significantly higher than that in B16-UV immunized mice and the control group (Figure 1b). In contrast, although there was no significant difference between the cytotoxicity in the SCC VII-NDV and SCC VII-UV immunized groups, the SCC VII-NDV immunized group significantly increased the cytotoxicity of tumor cells compared with the control group (Figure 1c). Previously, we also confirmed the cytotoxicity of tumor cells with WEHI164-NDV. The WEHI164-NDV group indicated significantly higher cytotoxicity compared with the WEHI164-UV and control groups. Furthermore, the level of cytotoxicity induced by WEHI-NDV was higher than that of immunization with B16-NDV and SCC VII-NDV. These results indicated that rNDV-TV induced an effective anti-tumor response. However, the difference in anti-tumor response, induced by rNDV-TV, was observed between each tumor cell line. Furthermore, it was suggested that it may be difficult for some tumor cell lines to induce sufficient cytotoxicity by NDV, because there was no significant difference between the cytotoxicity induced by SCC VII-NDV and SCC VII-UV.

It has been reported that NDV is more readily infects tumor cells than normal cells [1]. However, the difference in NDV infectivity between tumor cell lines has not been demonstrated until now. Then, to confirm the difference in susceptibility between tumor cell lines, the rNDV infection rate was examined in six kinds of tumor cell lines. The rNDV infection rate in each tumor cell line was as high as 80% in B16, WEHI 164, and 3LL. However, the infection rate was as low as 35% in SCC VII and MBT-2, and 13% in BALB-MC (Figure 2). These results indicated the difference in rNDV infectivity between tumor cell lines and suggested that rNDV infectivity might be related to the efficacy of TV-NDV. 

It has been reported that the difference in rNDV infectivity between tumor cells and normal cells is responsive to IFN-β [15], the gene expression of RIG-I, IRF-3, IRF-7, and IFN-β before infection [16] or the RIG-I gene and the receptor of type I IFN expression [17]. Therefore, Type I IFN-related gene expression was examined as a factor affecting infectivity of rNDV. Type I IFN-related gene expression, before infection, showed a significant negative correlation with the rNDV infection rate compared with those after infection (Table 1). Especially, OAS, OASL, Mx gene expression, and one of ISGs, which showed a significant negative correlation with rNDV infection. Consequently, it was suggested that ISG expression before infection affects rNDV susceptibility in tumor cells.

Furthermore, since the rNDV infectivity of the tumor cell lines showed a negative correlation with constitutive ISG expression, it was examined whether suppressing Janus kinases signal transducers, and activators of transcription (JAK-STAT) pathway, improves the rNDV infection rate in tumor cells. The rNDV infection rate was significantly improved in 6 tumor cell lines by Rux treatment suppressing JAK 1 and 2 (Figure 3). ISG expression after Rux treatment was significantly reduced and showed a significant negative correlation with the rNDV infection rate (Figure 4). These results revealed that the difference in rNDV infection rate in tumor cell lines was due to the degree of constitutive ISG expression, ISG expression decreased with JAK suppression, and the rNDV infection rate increased. Moreover, the finding that constitutive ISG expression, and the rNDV infection rate, show a negative correlation that might be useful as an indicator for application of viral therapy. This is a potential indicator because it is possible to predict the rNDV susceptibility in advance by confirmation of ISG expression in excised tumor tissue.

MBT-2 showed a low rNDV infection rate (Figure 2), suppression of ISG expression by Rux treatment (Figure 4), and an improved infection rate (Figure 3). Therefore, the immune response induced by rNDV-TV-Rux was examined in MBT-2. In MBT-NDV-Rux immunized mice, the number of CD8^+^ cells significantly increased in splenocytes seven days after immunization compared to the control and MBT-NDV groups (Figure 5b). Previously, we showed that immunized CD8^+^ cells are necessary for the anti-tumor response induced by rNDV-TV. The ratio of CD8^+^ and CD4^+^ cells were significantly increased in the mice transfused with natural killer T (NKT) cell, immunized CD8^+^ and CD4^+^ cells, and these mice survived compared with the mice transfused with NKT cell, non-immunized CD8^+^ and CD4^+^ cells [14]. Therefore, it was suggested that the induction of CD8^+^ cell growth by MBT-NDV-Rux might contribute to improvement of the anti-tumor response. Moreover, in IFN-γ gene expression in immunized splenocytes, the MBT-NDV-Rux immunized group significantly increased compared with the MBT-NDV immunized and control groups (Figure 6). These results suggest that the induction of the Th1 immune response was increased by the improvement of the rNDV susceptibility in tumor cells with Rux treatment. Previous reports indicated that infectious NDV produces more type I IFNs than inactivated NDV [18]. Type I IFN is considered an important factor in inducing tumor exclusion by rNDV-TV because type I IFN promotes maturation of dendritic cells and activates adaptive and innate immunity [4,5]. Virally derived HN protein from viruses expressed on the surface of NDV-infected cells induces antigen-specific cytotoxic activity by cytotoxic T cells (CTL) [19], and activates NK cells to induce IFN-γ and TNF-α production [6]. Furthermore, NH protein also induces IFN-α and TNF-α production from peripheral blood mononuclear cells [20]. Therefore, tumor cell susceptibility to rNDV s is considered a factor that improves rNDV-TV induced immune response.

This study shows that rNDV susceptibility in tumor cells affects the effect of the tumor immunotherapy with rNDV-TV. Moreover, by improving the rNDV susceptibility of tumor cells using the JAK inhibitor ruxolitinib, it was shown that the anti-tumor response induced by rNDV-TV occurs even in tumor cells with low susceptibility. These results suggest that rNDV-TV-Rux can induce an effective antitumor response, even in rNDV low-infectious tumors, and rNDV-TV-Rux may be a new strategy for tumor immune viral therapy.

## 5. Conclusions

In summary, it was revealed that rNDV infectivity differed depending on tumor cell lines, and it was found to be due to the difference in ISG expression before infection. Moreover, by using JAK inhibitor, ruxolitinib, we succeeded in suppressing ISG expression before infection and improving the rate of rNDV infection in tumor cells. Then, rNDV-TV-Rux, which improved rNDV infection rate in ruxolitinib, increased CD8 ^+^ cells important for inducing anti-tumor response by this tumor vaccine and induced a Th1 immune response compared to rNDV-TV. These findings are useful as a strategy for rNDV low-infectious tumors.

## Figures and Tables

**Table 1:** The correlation coefficient (r_s_) comparing the rNDV infection rate with type I interferon (IFN) related gene expression.

Gene	Pre-Infection	Post-Infection (8 h)
*RIG-I*	−0.480 *	−0.44
*TLR3*	−0.840 **	−0.486 *
*TLR7*	−0.342	−0.057
*IFN-β*	−0.139	−0.352
*IFN-α*	0.161	−0.028
*IRF-3*	−0.579 *	−0.347
*IRF-7*	−0.359	−0.594 *
*Mx1*	−0.518 *	−0.371
*Mx2*	−0.678 **	−0.304
*OAS1a*	−0.574 *	−0.373
*OAS1b*	−0.685 **	−0.411
*OAS2*	−0.612 **	−0.480
*OAS3*	−0.530 *	−0.373
*OASL1*	−0.417	−0.724 **
*OASL2*	−0.672 **	−0.637 **

Constitutive and rNDV-induced expression of type I IFN related genes analyzed in different tumor cell lines by qPCR. The correlation between rNDV infection rate and type I IFN related gene expression was calculated with the Spearman’s rank correlation coefficient (r_s_) and corresponding p-value (* *p* < 0.05, ** *p* < 0.01).

## Supplementary Materials

The following are available online at http://www.mdpi.com/2072-6694/10/6/186/s1, Figure S1: Constitutive and rNDV-induced expression of type I IFN related genes, Table S1: The list of primers, Table S2: The expression of type I IFN related genes in tumor cell lines.
