# Supplementary material for: Regulation of Constitutive Interferon-Stimulated Genes (Isgs) in Tumor Cells Contributes to Enhanced Antitumor Response of Newcastle Disease Virus-Infected Tumor Vaccines
Source: Cancers (Basel). 2018 Jun 6;10(6):186. doi: 10.3390/cancers10060186 (PMC6024929; doi:10.3390/cancers10060186)
Supplement: Supplementary file 1 [file cancers-10-00186-s001.pdf]

**Table S1.** The list of primers

| Gene | | Sequence of primer | Accession number |
|---|---|---|---|
| *GAPDH* | forward | 5′-CGTGAGTGGAGTCATACTGGAA-3′ | XM_017321385.1 |
| | reverse | 5′-AACGGATTTGGCCGTATTG-3′ | |
| *RIG-I* | forward | 5′-GCCCTGTACCATGCAGGTTAC-3′ | NM_172689.3 |
| | reverse | 5′-AGTCCCAACTTTCGATGGCTT-3′ | |
| *TLR3* | forward | 5′-TTGCGTTGCGAAGTGAAGAA-3′ | XM_006509283.3 |
| | reverse | 5′-ACTTGCCAATTGTCTGGAAACA-3′ | |
| *TLR7* | forward | 5′-AATCCACAGGCTCACCCATA-3′ | XM_011247786.1 |
| | reverse | 5′-CAGGTACCAAGGGATGTCCT-3′ | |
| *IRF-3* | forward | 5′-GTGCCTCTCCTGACACCAAT-3′ | XM_006540995.3 |
| | reverse | 5′-CCAAGATCAGGCCATCAAAT-3′ | |
| *IRF-7* | forward | 5′-AAGCTGGAGCCATGGGTATG-3′ | XM_006536214.1 |
| | reverse | 5′-GACCCAGGTCCATGAGGAAG-3′ | |
| *IFN-α* | forward | 5′-CTTCCACAGGATCACTGTGTACCT-3′ | NM_010505.2 |
| | reverse | 5′-TTCTGCTCTGACCACCTCCC-3′ | |
| *IFN-β* | forward | 5′-CTGGAGCAGCTGAATGGAAAG-3′ | NM_010510.1 |
| | reverse | 5′-CTTCTCCGTCATCTCCATAGGG-3′ | |
| *IFN-γ* | forward | 5′-TGAAAGCCTAGAAAGTCTGAATAAC-3′ | NM_008337.4 |
| | reverse | 5′-GTTGTTGCTGATGGCCTGAT-3′ | |
| *Mx1* | forward | 5′-CCAACTGGAATCCTCCTGGAA-3′ | NR_003520.1 |
| | reverse | 5′-GCCGCACCTTCTCCTCATAG-3′ | |
| *Mx2* | forward | 5′-CGAGAATTGCCAGGGTTTGT-3′ | NR_003508.1 |
| | reverse | 5′-GGCAGCTCGTACAATTTCAGT-3′ | |
| *OAS1a* | forward | 5′-GAAGAGGCTGATGTGTGGCT-3′ | XR_001784699.1 |
| | reverse | 5′-TGTCCAGTTCTCTTCTACCTGC-3′ | |
| *OAS1b* | forward | 5′-TTCTACGCCAATCTCATCAGTG-3′ | NR_003507.1 |
| | reverse | 5′-GGTCCCCCAGCTTCTCCTTAC-3′ | |
| *OAS2* | forward | 5′-AAACCTCACACCCAACGAAAA-3′ | XM_017320884.1 |
| | reverse | 5′-CCACCCTTAGCCACTTCCT-3′ | |
| *OAS3* | forward | 5′-CCAACCCAAGTGCCAATAAAA-3′ | NM_145226.2 |
| | reverse | 5′-AACATGACTTGAGATACTATCCAATGCT-3′ | |
| *OASL1* | forward | 5′-TGGACCTTGGGCTCAGTAAC-3′ | XM_006530295.3 |
| | reverse | 5′-GCACAACGGTGACAGTGATG-3′ | |
| *OASL2* | forward | 5′-CGATGCCTGGGAGAGAATCG-3′ | XM_006530315.3 |
| | reverse | 5′-TCGCCTGCTCTTCGAAACTG-3′ | |

**Table S2.** The expression of type I IFN related genes in tumor cell lines

(a)

| Normalized ratio | RIG-I | | TLR3 | | TLR7 | |
|---|---|---|---|---|---|---|
| | Pre-infection | Post-infection | Pre-infection | Post-infection | Pre-infection | Post-infection |
| B16-1 | 1.19E-03 | 0.06 | 9.24E-03 | 0.28 | 1.16E-03 | 9.71E-04 |
| B16-2 | 1.39E-03 | 0.04 | 8.16E-03 | 0.19 | 8.84E-04 | 1.11E-03 |
| B16-3 | 1.52E-03 | 0.05 | 0.01 | 0.16 | 7.35E-04 | 4.14E-03 |
| WEHI164-1 | 5.48E-03 | 0.11 | 0.01 | 0.27 | 5.63E-03 | 0.01 |
| WEHI164-2 | 6.69E-03 | 0.11 | 0.01 | 0.3 | 7.03E-03 | 9.80E-03 |
| WEHI164-3 | 9.25E-03 | 0.14 | 0.02 | 0.62 | 0.03 | 0.05 |
| 3LL-1 | 8.64E-03 | 0.04 | 2.44E-03 | 0.01 | 6.25E-03 | 0.02 |
| 3LL-2 | 7.40E-03 | 0.03 | 4.20E-03 | 0.01 | 5.24E-03 | 0.01 |
| 3LL-3 | 7.91E-03 | 0.01 | 2.24E-03 | 4.61E-03 | 3.89E-03 | 8.29E-03 |
| SCCVII-1 | 0.03 | 0.07 | 0.02 | 0.05 | 0.05 | 6.38E-03 |
| SCCVII-2 | 0.03 | 0.07 | 0.02 | 0.04 | 0.05 | 0.01 |
| SCCVII-3 | 0.02 | 0.08 | 3.90E-03 | 0.07 | 5.78E-03 | 0.1 |
| MBT-1 | 5.79E-03 | 0.04 | 0.02 | 0.08 | 4.08E-03 | 0.02 |
| MBT-2 | 8.77E-03 | 0.04 | 0.03 | 0.08 | 8.49E-03 | 0.05 |
| MBT-3 | 6.63E-03 | 0.06 | 0.03 | 0.09 | 3.04E-03 | 0.08 |
| BALB-1 | 0.05 | 0.17 | 0.14 | 0.74 | 0.02 | 6.36E-03 |
| BALB-2 | 0.04 | 0.12 | 0.1 | 1.07 | 0.05 | 2.78E-03 |
| BALB-3 | 0.04 | 0.15 | 0.08 | 1.31 | 7.22E-03 | 0.02 |
| calibrator | 1 | 1 | 1 | 1 | 1 | 1 |

(b)

| Normalized ratio | IFN-β | | IFN-α | | IRF-3 | |
|---|---|---|---|---|---|---|
| | Pre-infection | Post-infection | Pre-infection | Post-infection | Pre-infection | Post-infection |
| B16-1 | 0.02 | 0.5 | 0.04 | 0.19 | 0.02 | 0.03 |
| B16-2 | 0.04 | 0.95 | 0.24 | 0.38 | 8.56E-03 | 0.02 |
| B16-3 | 0.04 | 3.03 | 0.2 | 1.78 | 0.02 | 0.04 |
| WEHI164-1 | 0.05 | 12.95 | 0.15 | 2.84 | 0.03 | 0.07 |
| WEHI164-2 | 0.13 | 13.22 | 0.28 | 1.76 | 0.04 | 0.1 |
| WEHI164-3 | 7.44E-03 | 1.87 | 0.05 | | 0.05 | 0.05 |
| 3LL-1 | 0.12 | 2.15 | 0.25 | 1.67 | 0.03 | 0.05 |
| 3LL-2 | 0.04 | 0.81 | 0.14 | 0.64 | 0.03 | 0.04 |
| 3LL-3 | 0.13 | 0.13 | 0.33 | 0.07 | 0.04 | 0.03 |
| SCCVII-1 | 0.02 | 1.03 | 0.04 | 0.1 | 0.08 | 0.09 |
| SCCVII-2 | 8.70E-03 | 0.88 | 0.02 | 0.07 | 0.11 | 0.09 |
| SCCVII-3 | 0.12 | 0.86 | 0.15 | 0.05 | 0.08 | 0.13 |
| MBT-1 | 0.03 | 4.66 | 0.11 | 1.99 | 0.02 | 0.05 |
| MBT-2 | 0.35 | 2.29 | | 1.34 | 0.01 | 0.05 |
| MBT-3 | 0.09 | 19.7 | 0.18 | 26.42 | 0.02 | 0.04 |
| BALB-1 | 0.08 | 8.15 | 0.18 | | 0.09 | 0.07 |
| BALB-2 | 0.16 | 2.48 | 0.29 | 2.58 | 0.11 | 0.18 |
| BALB-3 | 0.85 | 5.88 | | 1.37 | 0.06 | 0.05 |
| calibrator | 1 | 1 | 1 | 1 | 1 | 1 |

(c)

| Normalized ratio | IRF-7 | | Mx1 | | Mx2 | |
|---|---|---|---|---|---|---|
| | Pre-infection | Post-infection | Pre-infection | Post-infection | Pre-infection | Post-infection |
| B16-1 | 2.52E-04 | 1.37E-03 | 2.38E-04 | 0.08 | 7.54E-03 | 5.39 |
| B16-2 | 3.15E-04 | 2.77E-03 | 2.04E-05 | 0.05 | | 3.18 |
| B16-3 | 9.48E-04 | 8.69E-03 | 1.08E-04 | 0.16 | 7.23E-03 | 2.95 |
| WEHI164-1 | 9.09E-04 | 0.04 | 1.40E-05 | 0.04 | 8.19E-03 | 4.74 |
| WEHI164-2 | 1.80E-03 | 0.04 | 1.01E-04 | 0.03 | | 4.58 |
| WEHI164-3 | 2.91E-04 | 0.02 | 3.84E-05 | 0.02 | 8.90E-03 | 6.83 |
| 3LL-1 | 9.71E-04 | 0.01 | 0 | 1.06E-04 | 4.05E-03 | |
| 3LL-2 | 7.68E-04 | 4.55E-03 | 2.68E-05 | 1.34E-04 | 2.19E-03 | 0.06 |
| 3LL-3 | 1.60E-03 | 1.22E-03 | 2.20E-05 | 3.45E-05 | | 0.13 |
| SCCVII-1 | 9.13E-04 | 9.52E-03 | 5.75E-04 | 9.22E-03 | 3.72E-03 | 3.88 |
| SCCVII-2 | 9.34E-04 | 6.95E-03 | 2.28E-04 | 3.06E-03 | 4.65E-03 | 2.85 |
| SCCVII-3 | 3.68E-03 | 0.01 | 4.75E-04 | 6.51E-03 | 1.05E-03 | 4.19 |
| MBT-1 | 8.79E-04 | 0.05 | 1.17E-04 | 4.12E-03 | 0.01 | 1.46 |
| MBT-2 | 6.87E-03 | 0.02 | 6.32E-05 | 2.61E-03 | 0.01 | 1.38 |
| MBT-3 | 1.48E-03 | | 0 | 2.43E-03 | | 0.01 |
| BALB-1 | 3.90E-03 | 0.1 | 3.12E-05 | 0.11 | 0.23 | 24.46 |
| BALB-2 | 4.29E-03 | 0.07 | 1.25E-03 | 0.35 | 0.17 | 16.75 |
| BALB-3 | 0.01 | 0.05 | 2.47E-04 | | 0.12 | 22.56 |
| calibrator | 1 | 1 | 1 | 1 | 1 | 1 |

(d)

| Normalized ratio | OAS1a | | OAS1b | | OAS2 | |
|---|---|---|---|---|---|---|
| | Pre-infection | Post-infection | Pre-infection | Post-infection | Pre-infection | Post-infection |
| B16-1 | 0.02 | 0.88 | 4.12E-03 | 3.36 | 0.17 | 1.92 |
| B16-2 | 0.02 | 0.5 | 2.86E-03 | 2 | 0.09 | 1.2 |
| B16-3 | | 0.58 | 9.93E-04 | 2.18 | 0.04 | 1.27 |
| WEHI164-1 | 0.05 | 0.55 | 0.03 | 5.54 | 0.25 | 1.86 |
| WEHI164-2 | | 0.53 | 0.02 | 4.44 | 0.11 | 1.99 |
| WEHI164-3 | 0.05 | 0.9 | 0.04 | 6.39 | 0.35 | 2.37 |
| 3LL-1 | 9.48E-03 | 0.06 | 0.05 | 0.56 | 0.32 | 0.52 |
| 3LL-2 | 0.02 | 0.05 | 0.05 | 0.25 | 0.43 | 0.54 |
| 3LL-3 | 6.37E-03 | 0.07 | 0.03 | 0.59 | 0.17 | 0.84 |
| SCCVII-1 | 0.02 | 0.36 | 0.06 | 2.61 | 0.73 | 1.77 |
| SCCVII-2 | 0.03 | 0.27 | 0.11 | 1.79 | 0.92 | 1.44 |
| SCCVII-3 | 0.02 | 0.49 | 0.05 | 2.45 | 0.46 | 1.29 |
| MBT-1 | 0.01 | 0.17 | 0.18 | 1.96 | 0.42 | 0.97 |
| MBT-2 | 0.01 | 0.09 | 0.07 | 2.2 | 0.2 | 1.57 |
| MBT-3 | 0.11 | 0.02 | | 0.18 | 1.37 | 0.65 |
| BALB-1 | 2.85 | 35.71 | 0.44 | 12.25 | 2.24 | 17.93 |
| BALB-2 | 2.99 | 27.07 | 0.47 | 8.87 | 2.12 | 15.76 |
| BALB-3 | 1.16 | 27.27 | 0.08 | 11.16 | 0.55 | 18.4 |
| calibrator | 1 | 1 | 1 | 1 | 1 | 1 |

(e)

| Normalized ratio | OAS3 | | OASL1 | | OASL2 | |
|---|---|---|---|---|---|---|
| | Pre-infection | Post-infection | Pre-infection | Post-infection | Pre-infection | Post-infection |
| B16-1 | 0.43 | 1.2 | 0.17 | 2.95 | 1.02E-05 | 6.67E-03 |
| B16-2 | 0.19 | 0.8 | 0.09 | 2.14 | 4.41E-06 | 4.48E-03 |
| B16-3 | 0.1 | 0.83 | 0.06 | 2.45 | 8.38E-06 | 0.01 |
| WEHI164-1 | 0.69 | 2.72 | 0.29 | 0.91 | 9.03E-05 | 0.04 |
| WEHI164-2 | 0.28 | 2.67 | 0.14 | 1.05 | 5.81E-05 | 0.03 |
| WEHI164-3 | 0.73 | 3.82 | 0.33 | 1.28 | 8.65E-05 | 0.02 |
| 3LL-1 | 0.79 | 1.12 | 0.84 | 2.24 | 2.41E-04 | 5.15E-03 |
| 3LL-2 | 1.02 | 0.81 | 1.15 | 1.81 | 3.72E-04 | 3.70E-03 |
| 3LL-3 | 0.47 | 1.72 | 0.57 | 2.84 | 2.59E-04 | 1.71E-03 |
| SCCVII-1 | 1.86 | 3.27 | 1.1 | 6.93 | 7.77E-04 | 0.03 |
| SCCVII-2 | 2.43 | 2.6 | 1.48 | 5.45 | 6.73E-04 | 0.03 |
| SCCVII-3 | 1.35 | 2.37 | 0.69 | 6.41 | 8.29E-04 | 0.03 |
| MBT-1 | 0.97 | 1.09 | 0.65 | 4.39 | 1.17E-03 | 0.05 |
| MBT-2 | 0.52 | 1.5 | 0.34 | 4.12 | 1.08E-03 | 0.03 |
| MBT-3 | 1.34 | 1.5 | 1.62 | 1.71 | 1.08E-03 | 0.01 |
| BALB-1 | 2.93 | 4.74 | 1.71 | 22.08 | 1.91E-03 | 0.06 |
| BALB-2 | 3.05 | 3.6 | 1.45 | 16.99 | 1.58E-03 | 0.2 |
| BALB-3 | 0.85 | 5.13 | 0.62 | 19.88 | 1.84E-03 | 0.02 |
| calibrator | 1 | 1 | 1 | 1 | 1 | 1 |

Constitutive and rNDV-induced expression of type I IFN related genes analyzed in different tumor cell lines by qPCR. The gene expression of (a)RIG-I, TLR3,TLR7, (b)IFN-β, IFN-α, IRF-3, (c)IRF-7, Mx1, Mx2, (d)OAS1a, OAS1b, OAS2, (e)OAS3, OASL1, OASL2 were measured as type I IFN related genes. The gene expression was normalized by calibrator.

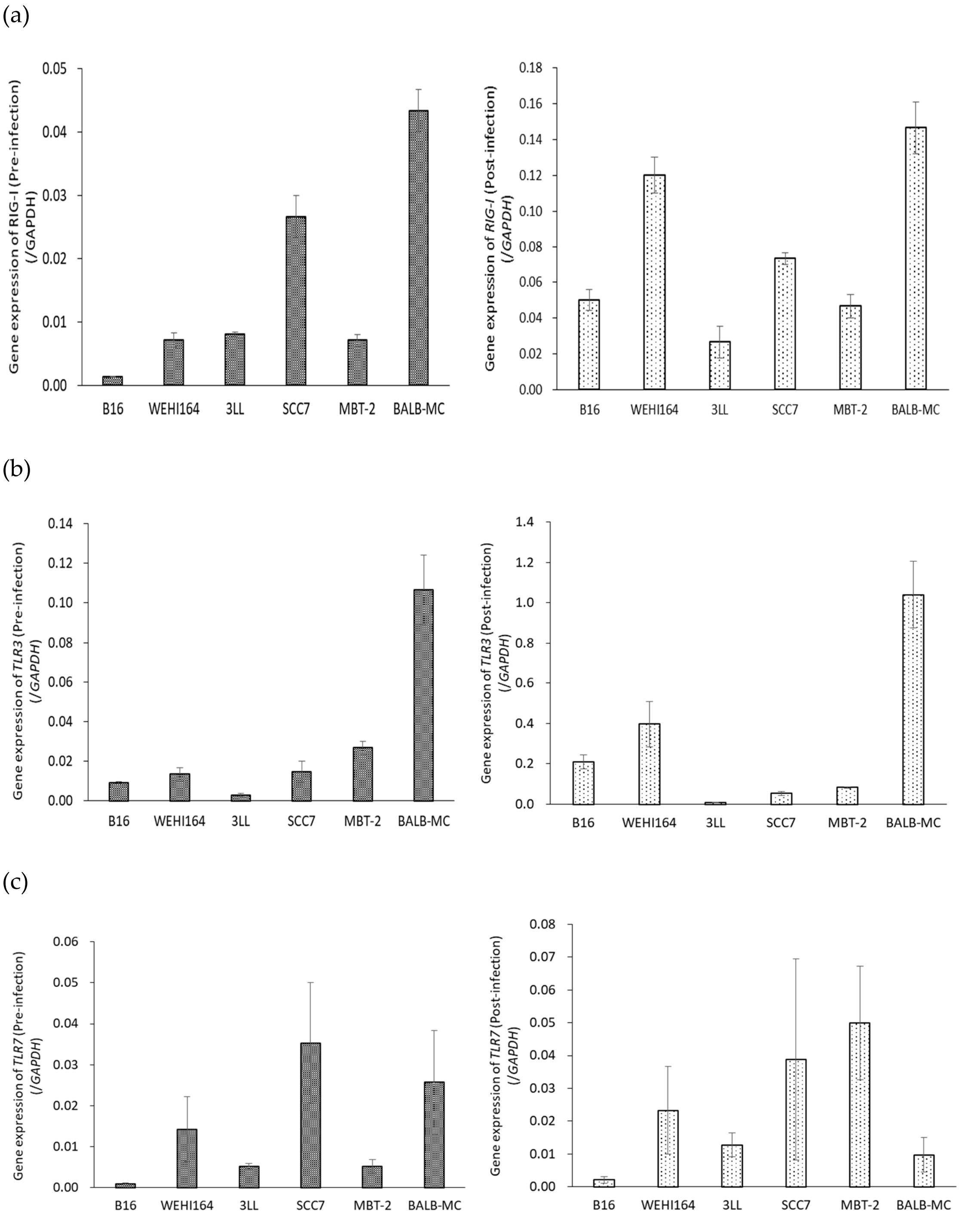

(a)
Gene expression of RIG-I (Pre-infection) (/GAPDH)
0.00 0.01 0.02 0.03 0.04 0.05
Gene expression of RIG-I (Post-infection) (/GAPDH)
0.00 0.02 0.04 0.06 0.08 0.10 0.12 0.14 0.16 0.18
(b)
Gene expression of TLR3 (Pre-infection) (/GAPDH)
0.00 0.02 0.04 0.06 0.08 0.10 0.12 0.14
Gene expression of TLR3 (Post-infection) (/GAPDH)
0.0 0.2 0.4 0.6 0.8 1.0 1.2 1.4
(c)
Gene expression of TLR7 (Pre-infection) (/GAPDH)
0.00 0.01 0.02 0.03 0.04 0.05 0.06
Gene expression of TLR7 (Post-infection) (/GAPDH)
0.00 0.01 0.02 0.03 0.04 0.05 0.06 0.07 0.08
B16
WEHI164
3LL
SCC7
MBT-2
BALB-MC

(d)

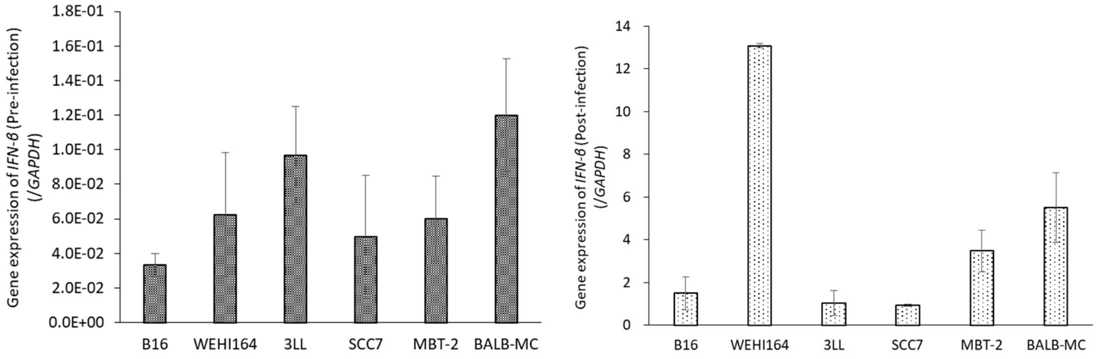
Gene expression of IFN-β (Pre-infection) (/GAPDH)
1.8E-01
1.6E-01
1.4E-01
1.2E-01
1.0E-01
8.0E-02
6.0E-02
4.0E-02
2.0E-02
0.0E+00
B16
WEHI164
3LL
SCC7
MBT-2
BALB-MC
Gene expression of IFN-β (Post-infection) (/GAPDH)
14
12
10
8
6
4
2
0
B16
WEHI164
3LL
SCC7
MBT-2
BALB-MC

(e)

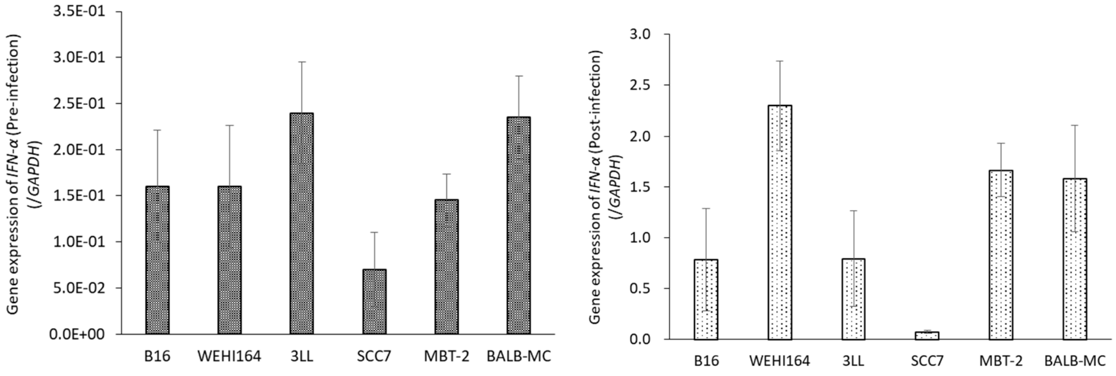
Gene expression of IFN-α (Pre-infection) (/GAPDH)
3.5E-01
3.0E-01
2.5E-01
2.0E-01
1.5E-01
1.0E-01
5.0E-02
0.0E+00
B16
WEHI164
3LL
SCC7
MBT-2
BALB-MC
Gene expression of IFN-α (Post-infection) (/GAPDH)
3.0
2.5
2.0
1.5
1.0
0.5
0.0
B16
WEHI164
3LL
SCC7
MBT-2
BALB-MC

(f)

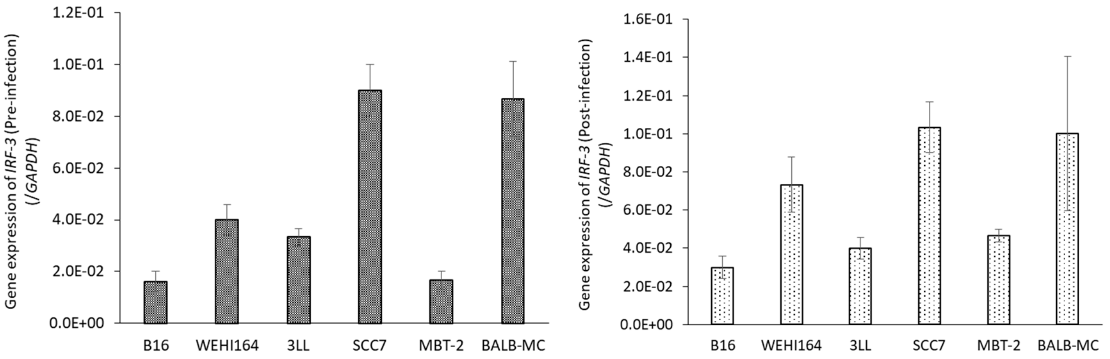
Gene expression of IRF-3 (Pre-infection) (/GAPDH)
1.2E-01
1.0E-01
8.0E-02
6.0E-02
4.0E-02
2.0E-02
0.0E+00
B16
WEHI164
3LL
SCC7
MBT-2
BALB-MC
Gene expression of IRF-3 (Post-infection) (/GAPDH)
1.6E-01
1.4E-01
1.2E-01
1.0E-01
8.0E-02
6.0E-02
4.0E-02
2.0E-02
0.0E+00
B16
WEHI164
3LL
SCC7
MBT-2
BALB-MC

(g)

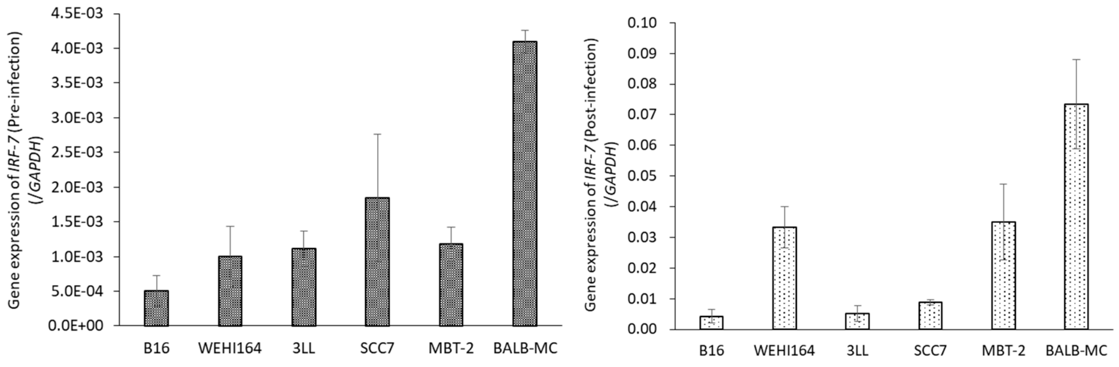
Gene expression of IRF-7 (Pre-infection) (/GAPDH)
4.5E-03
4.0E-03
3.5E-03
3.0E-03
2.5E-03
2.0E-03
1.5E-03
1.0E-03
5.0E-04
0.0E+00
B16
WEHI164
3LL
SCC7
MBT-2
BALB-MC
Gene expression of IRF-7 (Post-infection) (/GAPDH)
0.10
0.09
0.08
0.07
0.06
0.05
0.04
0.03
0.02
0.01
0.00
B16
WEHI164
3LL
SCC7
MBT-2
BALB-MC

(h)

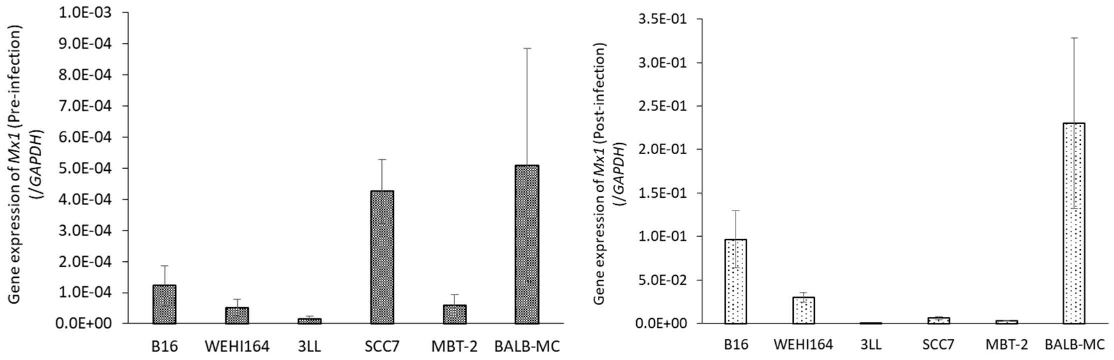
Gene expression of Mx1 (Pre-infection) (/GAPDH)
1.0E-03
9.0E-04
8.0E-04
7.0E-04
6.0E-04
5.0E-04
4.0E-04
3.0E-04
2.0E-04
1.0E-04
0.0E+00
B16
WEHI164
3LL
SCC7
MBT-2
BALB-MC
Gene expression of Mx1 (Post-infection) (/GAPDH)
3.5E-01
3.0E-01
2.5E-01
2.0E-01
1.5E-01
1.0E-01
5.0E-02
0.0E+00
B16
WEHI164
3LL
SCC7
MBT-2
BALB-MC

(i)

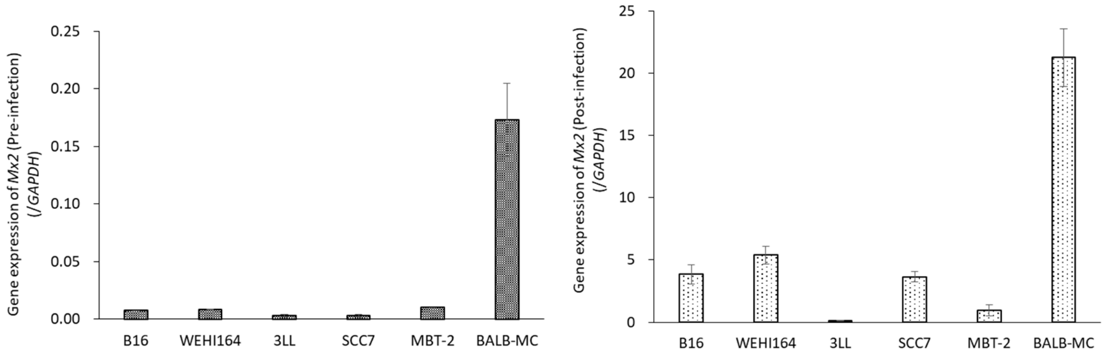
Gene expression of Mx2 (Pre-infection) (/GAPDH)
0.25
0.20
0.15
0.10
0.05
0.00
B16
WEHI164
3LL
SCC7
MBT-2
BALB-MC
Gene expression of Mx2 (Post-infection) (/GAPDH)
25
20
15
10
5
0
B16
WEHI164
3LL
SCC7
MBT-2
BALB-MC

(j)

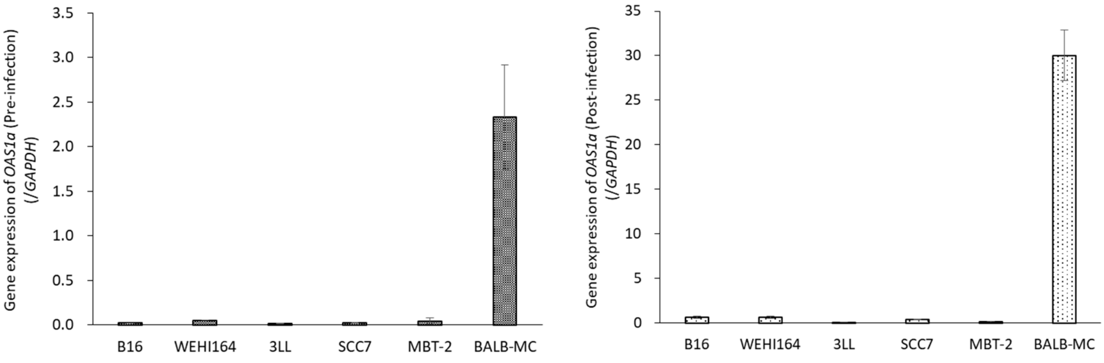
Gene expression of OAS1a (Pre-infection) (/GAPDH)
3.5
3.0
2.5
2.0
1.5
1.0
0.5
0.0
B16
WEHI164
3LL
SCC7
MBT-2
BALB-MC
Gene expression of OAS1a (Post-infection) (/GAPDH)
35
30
25
20
15
10
5
0
B16
WEHI164
3LL
SCC7
MBT-2
BALB-MC

(k)

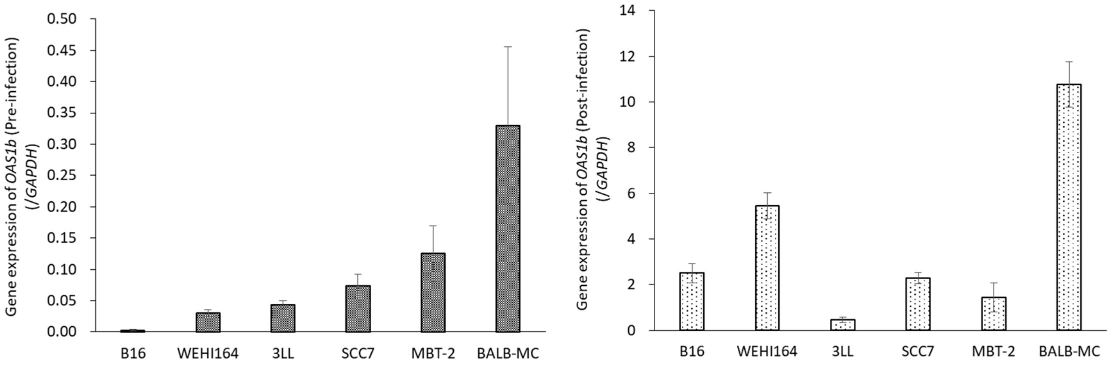
Gene expression of OAS1b (Pre-infection) (/GAPDH)
0.00
0.05
0.10
0.15
0.20
0.25
0.30
0.35
0.40
0.45
0.50
B16
WEHI164
3LL
SCC7
MBT-2
BALB-MC
Gene expression of OAS1b (Post-infection) (/GAPDH)
0
2
4
6
8
10
12
14
B16
WEHI164
3LL
SCC7
MBT-2
BALB-MC

(l)

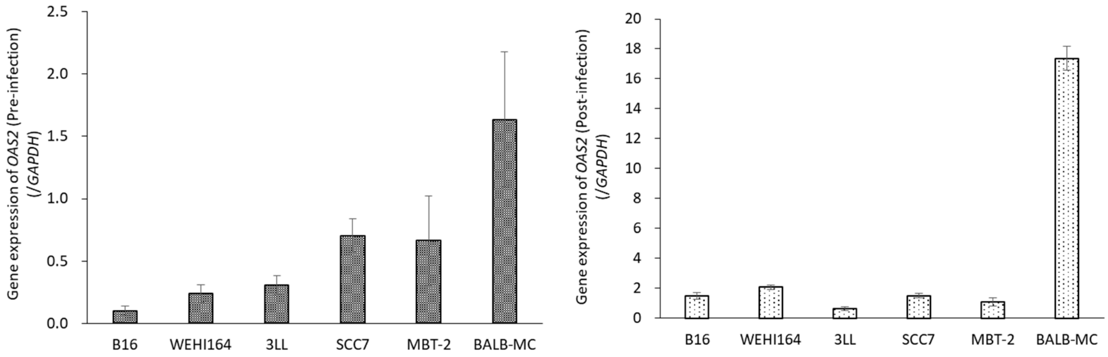
Gene expression of OAS2 (Pre-infection) (/GAPDH)
0.0
0.5
1.0
1.5
2.0
2.5
B16
WEHI164
3LL
SCC7
MBT-2
BALB-MC
Gene expression of OAS2 (Post-infection) (/GAPDH)
0
2
4
6
8
10
12
14
16
18
20
B16
WEHI164
3LL
SCC7
MBT-2
BALB-MC

(m)

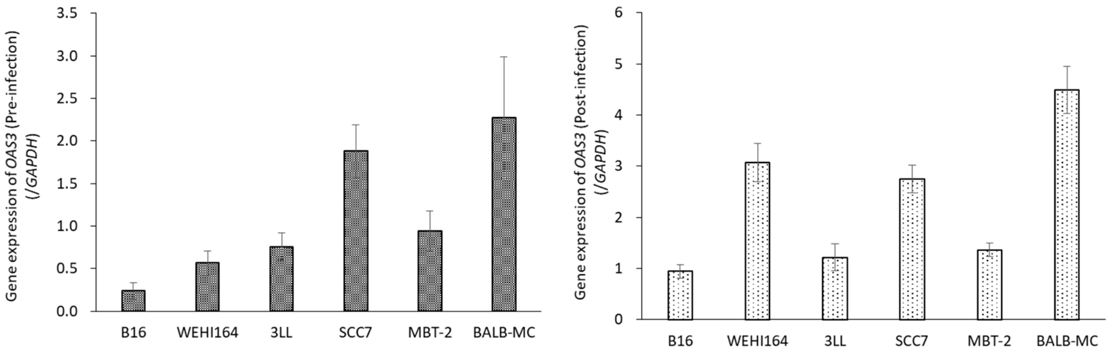
Gene expression of OAS3 (Pre-infection) (/GAPDH)
0.0
0.5
1.0
1.5
2.0
2.5
3.0
3.5
B16
WEHI164
3LL
SCC7
MBT-2
BALB-MC
Gene expression of OAS3 (Post-infection) (/GAPDH)
0
1
2
3
4
5
6
B16
WEHI164
3LL
SCC7
MBT-2
BALB-MC

(n)

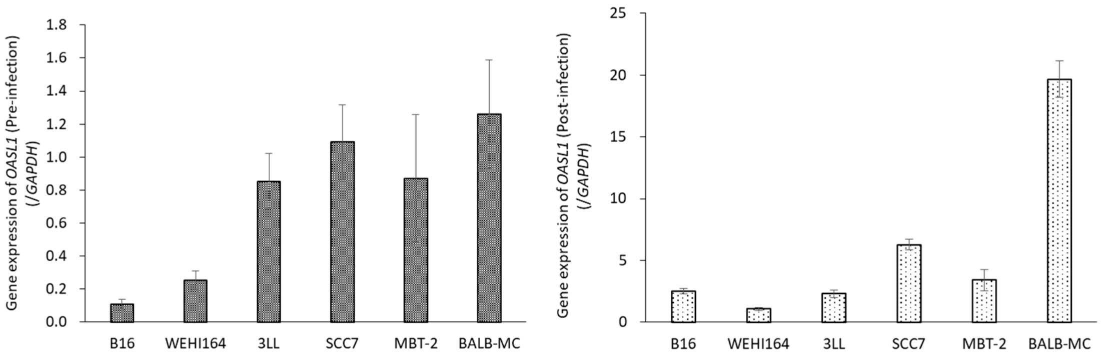
Gene expression of OASL1 (Pre-infection) (/GAPDH)
0.0
0.2
0.4
0.6
0.8
1.0
1.2
1.4
1.6
1.8
B16
WEHI164
3LL
SCC7
MBT-2
BALB-MC
Gene expression of OASL1 (Post-infection) (/GAPDH)
0
5
10
15
20
25
B16
WEHI164
3LL
SCC7
MBT-2
BALB-MC

(o)

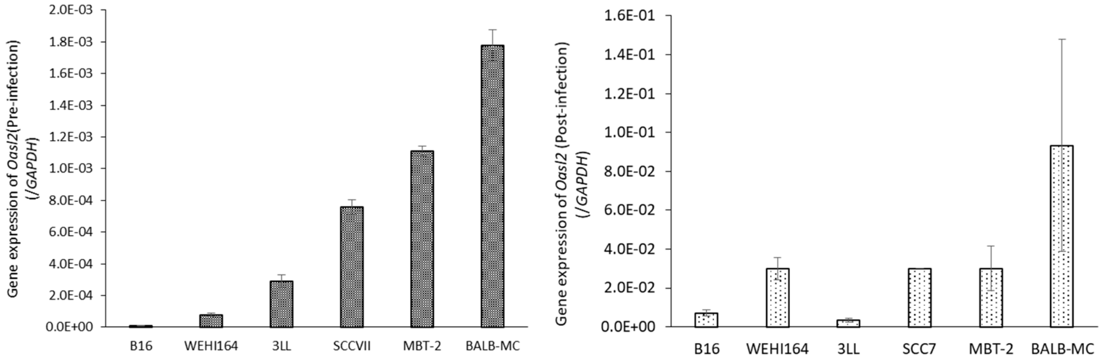


Figure S1. Constitutive and rNDV-induced expression of type I IFN related genes. (a)RIG-I, (b)TLR3, (c)TLR7, (d)IFN-β, (e)IFN-$\alpha$, (f)IRF-3, (g)IRF-7, (h)Mx1, (i)Mx2, (j)OAS1a, (k)OAS1b, (l)OAS2, (m)OAS3, (n)OASL1 and (o)OASL2 were measured as type I IFN related genes by qPCR (left: pre-infection, right: post-8h infection). Gene expression was normalized with GAPDH.
